# 

**Published:** 1894-01-20

**Authors:** 


CONTENTS.

**Sir Andrew Clark: A Monument** - 253
**Around the Hospitals** - 254
**Treatment of Poisoning by Chloral Hydrate.** By Sir BENJAMIN WARD RICHARDSON, M.D., F.R.S. - 255
**Art and Disease.** II.—Possession or Hysteria (*continued*) - 256
**Medical Progress and Hospital Clinics—**
A Plaster Bed in the Treatment of Pott's Disease - 261
Drainage in Surgical Treatment - 262
NEW APPLIANCES AND THINGS MEDICAL.—Germ White Bread - 262
MEDICAL PROGRESS—Liver and Gall Bladder - 263
Pulmonary Tuberculosis (*continued*) - 264
Ophthalmology - 266
The Serum and Antitoxin Treatment of Tetanus.—Salophen - 268
**Diseases of the Brain Due to Diseases of the Ear** - 257
**The Field of Science** - 258
**Annotations.**—Some Dangers of Self Drugging—Albuminura and Life Assurance—Should Midwives be Registered? - 259
**Notes and News** - 260
**The Institutional Workshop—**
HOSPITAL ADMINISTRATION.—Hospital Abuse and the Working Class - 269
The Poor in London - 270
PRACTICAL DEPARTMENTS—Appliances at Poplar (*continued*) - 271
CONSTRUCTION NOTES - 272
**Editor's Letter-Box.**—Midwives Registration Association - 272
**Scraps and Gleanings** - 272

EXTRA SUPPLEMENT.—THE NURSING MIRROR.

**News from the Nursing World.**—Our Princess—A Royal Example—Dangerous Decorations—Sad Realities—Work Without Wages—Without Training or Experience—Significant Symptoms—A Precarious Livelihood—A Nurse's Responsibility—Vancouver—Benares—Cupboards—Short Items - cli
**On Nursing the Nervous and Insane.** II.—Diseases of the Nervous System - cliii
**District Nursing.**—III. Practical Work - cliv
**Canadian Notes—Minor Appointments** - clv
**Nursing in Paris.**—L'Hospice de la Maternité - clvi
**Women's Work.**—The Responsibilities of Manufacturers' Wives - clvi
**The After-Care Association** - clvii
**Death by Misadventure** - clvii
**The Prevention of Consumption Amongst the Poor** - clviii

